# Sharp detection of oscillation packets in rich time-frequency representations of neural signals

**DOI:** 10.3389/fnhum.2023.1112415

**Published:** 2023-12-07

**Authors:** Eugen-Richard Ardelean, Harald Bârzan, Ana-Maria Ichim, Raul Cristian Mureşan, Vasile Vlad Moca

**Affiliations:** ^1^Experimental and Theoretical Neuroscience Laboratory, Transylvanian Institute of Neuroscience, Cluj-Napoca, Romania; ^2^Computer Science Department, Technical University of Cluj-Napoca, Cluj-Napoca, Romania; ^3^STAR-UBB Institute, Babeş-Bolyai University, Cluj-Napoca, Romania

**Keywords:** neural oscillations, burst, detection, quantification, time-frequency spectrum, superlets

## Abstract

Brain oscillations most often occur in bursts, called oscillation packets, which span a finite extent in time and frequency. Recent studies have shown that these packets portray a much more dynamic picture of synchronization and transient communication between sites than previously thought. To understand their nature and statistical properties, techniques are needed to objectively detect oscillation packets and to quantify their temporal and frequency extent, as well as their magnitude. There are various methods to detect bursts of oscillations. The simplest ones divide the signal into band limited sub-components, quantifying the strength of the resulting components. These methods cannot by themselves cope with broadband transients that look like genuine oscillations when restricted to a narrow band. The most successful detection methods rely on time-frequency representations, which can readily show broadband transients and harmonics. However, the performance of such methods is conditioned by the ability of the representation to localize packets simultaneously in time and frequency, and by the capabilities of packet detection techniques, whose current state of the art is limited to extraction of bounding boxes. Here, we focus on the second problem, introducing two detection methods that use concepts derived from clustering and topographic prominence. These methods are able to delineate the packets’ precise contour in the time-frequency plane. We validate the new approaches using both synthetic and real data recorded in humans and animals and rely on a super-resolution time-frequency representation, namely the superlets, as input to the detection algorithms. In addition, we define robust tests for benchmarking and compare the new methods to previous techniques. Results indicate that the two methods we introduce shine in low signal-to-noise ratio conditions, where they only miss a fraction of packets undetected by previous methods. Finally, algorithms that delineate precisely the border of spectral features and their subcomponents offer far more valuable information than simple rectangular bounding boxes (time and frequency span) and can provide a solid foundation to investigate neural oscillations’ dynamics.

## 1 Introduction

Neural computations and information transmission in the brain are accompanied by oscillations (Wang, 2010) embedded in rich time-frequency landscapes (Moca et al., 2021; Bârzan et al., 2022). Oscillations often appear as events of finite duration and finite frequency span, called oscillation bursts or packets, intermixed with sustained oscillations and transient broadband events (Tal et al., 2020). For example, bursts of gamma (30–80 Hz) have been found to be modulated by attention in the cat auditory cortex (Lakatos et al., 2004), to subserve memory encoding and retrieval in monkeys (Lundqvist et al., 2016), alongside beta bursts (12–30 Hz), and to transiently couple distant areas in human electroencephalogram (EEG) during conscious perception (Melloni et al., 2007; Hipp et al., 2011). Moreover, bursts of oscillations have been found virtually in all relevant frequency bands [see (Tal et al., 2020) for a review]. As the authors note, even in Berger’s first historical account of an oscillation (Berger, 1930), alpha (8–12 Hz) are crayoned as transient events.

In addition to oscillation bursts, there are other features of interest in spectra of neuronal signals. Broadband shifts in frequencies above 80 Hz (Manning et al., 2009; Waldman et al., 2018) have been shown to correlate with increased neuronal firing (Buzsáki, 1986; Manning et al., 2009; Ray and Maunsell, 2011), to reflect the balance between excitation and inhibition (Gao et al., 2017), and to correlate with behavioral task performance (Honey et al., 2012; Miller et al., 2014). Pathologic epileptic activity is characterized by a combination of oscillations and brief spike discharges events (sharp waves), with high power in a wide bandwidth (Crépon et al., 2010; Staba et al., 2014; Park et al., 2020). Given the rich content of biological spectra comprising not only oscillations, but also features such as high-frequency shifts, and brief broadband events of neuronal or artifactual origin, it is crucial to develop methods able to localize and describe the shape of these features. Consequently, their properties, such as shape, power, frequency, duration, etc. that reflect the underlying processes (Buzsaki, 2006), could be quantified precisely. Here we introduce two methods able to detect and delineate precisely the spectral features (or packets) of arbitrary spectral shapes and over wide frequency bands.

Detection of packets is difficult due to methodological limitations: a robust detection method applicable over wide frequency bands has not been developed yet. In recent years, the increased awareness over the oscillations’ finite, burst-like expression, has led to the development of a number of methods for detection. Based on the applicability domain these detection methods can be divided into two main categories, namely: (i) those developed and optimized for high-frequency oscillations (HFOs) and, (ii) methods intended to detect oscillation packets in a larger context and not tunned for HFOs specifically. In the following, we briefly introduce these methods and their main traits.

The first category comprises methods optimized specifically for HFOs, which are applied in the context of epilepsy. One thing to note is that, HFOs is a rather loose term (Staba et al., 2014) that covers different sub bands, usually above 90 Hz (Buzsáki and Draguhn, 2004; Buzsáki and da Silva, 2012; Staba et al., 2014), but see Gardner et al. (2007) who terms a sub-gamma range (40–100 Hz) as HFOs. Most of these detection algorithms filter out data outside the HFOs sub-band of interest, and then use some measure of energy (Staba et al., 2002; Worrell et al., 2008; Zelmann et al., 2010, 2012), or the Hilbert transform (Crépon et al., 2010) to detect HFOs based on thresholds derived from the statistical properties of the data. Subsequently HFOs are checked against epilepsy-specific criteria such as duration or repetition, sometimes under the assumption that HFOs are rare events (Zelmann et al., 2012). Surprisingly, what is considered the gold standard of HFO detection by some authors (Donos et al., 2020) is based on the visual inspection of filtered signal traces (Jacobs et al., 2009), which is prone to subjectivity and errors. In brief, periods where the 80 Hz high-pass filtered signal shows increased activity are considered HFOs only if their spectra do not extend beyond 250 Hz. Consequently, HFOs are validated when there is no concomitant increase in the signal filtered with a high-pass 250 Hz filter. In a more recent paper (Donos et al., 2020) a radically different approach is taken. The authors use computer vision algorithms in an iterative manner to detect blobs of oscillations in wavelet time-frequency representations (TFRs). As with previous methods, detected blobs are validated against HFO’s criteria. Another interesting approach (Waldman et al., 2018) uses the iso-power contours in wavelet spectra to identify ripple on spike (RonS) HFOs. A wavelet TFR, limited to the ripple band (80–250 Hz), is computed using time-domain convolution. A “blob” (or packet) with increased power in the ripple band is identified as possible HFO only if its contours are closed, that is, if the packet is fully contained within the band. Next, the RonSs are validated only if the timing of ripple waveform (isolated with an 80 Hz high-pass filter) matches the timing of the raw signal. While interesting and diverse in their approach, HFO detectors are heavily specialized on epilepsy applications and are difficult to readily transfer to the broader range of neural oscillations especially in the area below 80 Hz.

The second category of methods, designed to detect oscillations across the whole relevant spectrum, has been recently reviewed (Tal et al., 2020). Here, we only briefly discuss different classes of algorithms to highlight their most important advantages and shortcomings. A first class of algorithms looks for oscillations in well-defined frequency bands either by thresholding the energy (Caplan et al., 2001), or by looking for peaks in the power of the dominant frequency within the band (Sherman et al., 2016). All these algorithms are confined within the limits of the chosen frequency bands. Due to their simple approach, they are unable to resolve fine time-frequency structures or to deal with broadband packets but have been used successfully for real-time closed-loop experiments (Karvat et al., 2020).

Another class of algorithms operates on TFRs. Threshold algorithms within this category derive distinct thresholds for each frequency either from the statistics of the individual frequencies (Lundqvist et al., 2016), or from a 1/*f* model of the data (Hughes et al., 2012). While these algorithms can detect oscillation bursts at any frequency, they are usually tuned around a subset of frequencies where oscillations’ occurrence has been established, for instance from the averaged power spectra. The concept of rhythmicity or phase consistency in time was used to recover small amplitude oscillations (Fransen et al., 2015). The authors show that lagged coherence can better uncover distinct oscillations than Fourier TFRs. Since phase stability only makes sense for one given frequency, the method is not well suited for more complex time-frequency patterns. This is the case for any algorithm that operates per one frequency basis. To the best of our knowledge, rhythmicity has been used in the alpha and beta bands and has not been validated on higher frequencies. An interesting approach that employs Hidden Markov Models (Quinn et al., 2019) was able to detect complex spatiotemporal structures in the TFRs. However, this algorithm does not operate on high frequencies (> 48 Hz) either, where oscillations are difficult to detect. Another difficulty lies with the number of hidden states, an important parameter whose choice is not clear. Similar to HFOs, many of the algorithms mentioned above require the detected burst to contain at least a minimum number of cycles.

The most versatile TFR-based algorithm to date is OEvents (Neymotin et al., 2022). It operates not on individual frequencies but rather on the whole time-frequency plane where it detects oscillation bursts as local maxima. Bursts span over time and frequency to an extent defined by per-frequency thresholds. Finally, each burst is localized within a rectangular bounding box and is quantified by a set of parameters such as, extent, number of cycles, dominant frequency, and power, etc. In this particular instance, the wavelet TFR was used, and frequencies were equalized in a pre-processing step, which is one of the main strengths of the algorithm and the reason why the algorithm was able to operate up to 200 Hz. This is by far the most successful algorithm to date and has been used to characterize oscillation bursts in intracranial recordings from humans and monkeys. Nevertheless, there is room for improvement. One of the downsides of OEvents is that the fine spatial structures of the oscillations are lost. Rectangular bounding boxes fail to properly characterize the rich repertoire of shapes found in TFRs. Another weakness stems from the properties of the TFR of choice. Wavelets, although well localized in time, struggle with resolution in high frequencies (Moca et al., 2021), a weakness that is inherently transferred to OEvents, which makes the algorithm overall less suited to process broad frequency ranges (i.e., from delta to high gamma).

Finally, another class of algorithms uses a two-step approach. First, oscillations are detected by power thresholds determined in the frequency domain. Next, stereotypical waveforms are sought for bursts, under the assumption that the waveform reflects properties of the generating substrate. Although interesting, these algorithms fall outside the scope of this paper and have been reviewed previously in Tal et al. (2020).

Although proper detection and quantification of oscillation packets is important, so far this has been hindered by the limitations of detection methods. Detection methods that rely on TFRs are further limited by the ability of the TFRs to represent oscillation packets scattered over the time-frequency landscape. In the next sections, we define two new detection techniques, which are able to precisely extract the contour of the estimated packets. To validate the two packet detection algorithms introduced here, we will evaluate their performance on well-established TFRs, namely the Short-Time Fourier Transform (STFT) and the continuous wavelet transform (CWT), as well as on super-resolution TFRs computed with superlets (Moca et al., 2021). The latter are exceptionally good at revealing the presence of packets in complex time-frequency spectra of neural signals. The most complex of the detection methods we introduce here is able to find oscillations with high success rates even in scenarios with low signal-to-noise ratio (SNR), where simpler threshold-based algorithms fail. Overall, we show that complex algorithms are better at capturing the structure of oscillation bursts because contours and sub-peaks reveal far more about the TFR structure than simple bounding boxes and can be instrumental in probing single-trial oscillation dynamics.

## 2 Materials and equipment

### 2.1 *In vivo* electrophysiology

Adult C57/BL6J mice were anesthetized using isoflurane (5% for induction, 2–2.5% for surgery) and then mounted in a stereotaxic frame (Stoelting Co, IL, United States). The animal’s body temperature was monitored and maintained at 37°C using a feedback-controlling heating pad with a rectal probe (Harvard Apparatus, MA, United States). The head of the animal was shaved and prepped with povidone-iodine and a local anesthetic (Xylocaine). Following a midline incision, a circular 2 mm craniotomy was performed on the left hemisphere targeting stereotaxic coordinates corresponding to the visual cortex (0.5–1 mm anterior from lambda, 2–2.5 mm lateral from midline). A 32-channel silicon probe (Cambridge NeuroTech, Cambridge, United Kingdom) was mounted on the stereotaxic manipulator and slowly inserted into the brain.

The electrophysiological signals were acquired at a sampling frequency of 32 kHz (Multi Channel Systems GmbH, Reutlingen, Germany) and local field potentials (LFPs) were obtained by band-pass filtering (Butterworth IIR filter, bidirectional, 3rd order, 0.1–300 Hz) and downsampling to 1 kHz. Line noise artifacts and their harmonics were removed using a series of notch filters (Butterworth IIR filter, 3rd order, bidirectional @50, 100, 150 Hz).

### 2.2 Electroencephalography

The EEG dataset used in this study was collected from healthy human subjects using a high-density EEG cap (Biosemi ActiveTwo, Amsterdam, Netherlands) consisting of 128 electrodes and recorded at a sampling rate of 1,024 Hz during a visual recognition task. Visual stimuli were generated with the “Dots” method (Moca et al., 2011) and presented under a viewing angle of 8.7 × 5.6 on a 22-inch Samsung SyncMaster 226BW LCD monitor with a resolution of 1,480 × 11,050 @ 120 fps positioned 1.12 m in front of the subject. Subsequently, EEG data was band-pass filtered to 0.1–200 Hz (Butterworth 3rd order) and the power line noise was rejected with a band stop filter (49.5–50.5 Hz, 4th order Butterworth). Both filters were applied bidirectionally for zero phase distortions.

We used a collection of 210 stimuli, consisting of dot lattices deformed progressively to resemble the contours of 30 familiar objects. Each trial had 3 intervals: fixation, stimulation, and response. In the fixation interval, each participant was instructed to fixate for 1,500–2,000 ms (baseline period) before the stimulus appeared on the monitor. Following the stimulus presentation, the participant was free to explore the visual scene for as much as needed to reach a perceptual decision regarding the identity of the stimulus. The trial ended with the response interval, where each participant had to respond by pressing one of the three buttons corresponding to the perceptual decision: seen, uncertain, and nothing.

## 3 Methods

Here we introduce two methods that segment time-frequency representations (TFRs) into regions of interest (ROIs) defined by power, namely the time-frequency breakdown method (TFBM) and the time-frequency peak finder (TFPF). Both algorithms are designed to identify areas with increased power levels that stand out from their neighbors in the TFR. The boundaries are not just simple bounding boxes, but reflect in detail the contours that encompass their corresponding peak. Both algorithms group together, in the same ROI, peaks that are poorly separated, under the assumption that these could belong to the same process. There is no other prior assumption on the detected ROIs and their characteristics. They can be oscillations with a clear dominant frequency (bursts or sustained), broadband events such as avalanches or artifacts, or could be an “exotic” superposition of contributions from various sources. In the following, both algorithms are described in detail.

### 3.1 Detection of oscillation packets with TFBM

#### 3.1.1 Core principle

**FIGURE 1:**
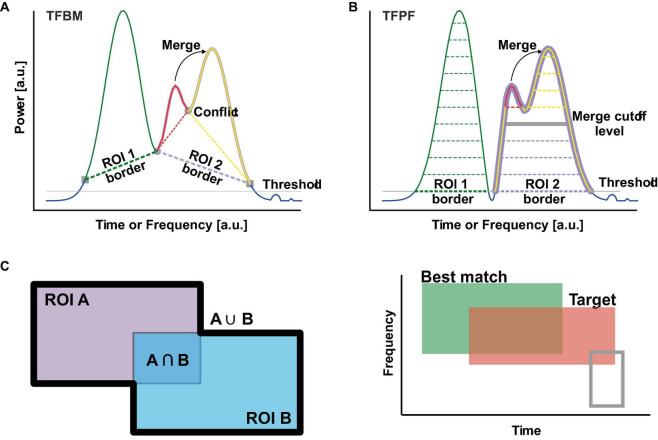
Segmentation algorithms and ROI matching metric. **(A)** To segment the TFR in ROIs, TFBM starts at peaks from where it follows a descending path until it encounters a conflict point (gray circle) or a point that does not meet the expansion criteria (gray squares). Next, the algorithm resolves conflicts by assigning the points of conflict to one of the ROIs or by merging the conflicting ROIs into a larger ROI, whose border is depicted with dotted lines. Peaks that are below the threshold (thin gray line) are not considered as seeds for ROI expansion. **(B)** TFPF slices through the TFR from the highest power to the threshold (gray line). ROIs (dotted lines) expand as the algorithm slice level is lowered. When ROIs merge (thick gray line) the smaller peak is merged into the larger one. The algorithm stops at the threshold (thing gray line). Points below the threshold are not taken into consideration. **(C)** The left pane illustrates the matching metric between two ROIs. On the right, best matching ROI (green) overlaps with the target ROI (red). The gray ROI has a lower match value to the target than the best matching ROI.

Time-frequency breakdown method (TFBM) (Figure 1A) traverses an intensity map, namely the TFR, searching for ROIs with a clear increase in power. At first, TFBM looks for potential peaks of oscillations by searching for local maxima that become packet center candidates. All candidates below a certain threshold (see section “3.1.2 Validation of local maxima”) are discarded in order to avoid very small fluctuations that usually result in a large number of spurious noisy peaks with small amplitudes near the floor of the TFR. Once valid candidate peaks are identified, a modified breadth-first search (BFS) algorithm expands each of the peaks iteratively into ROIs. The peaks with higher power values take precedence during expansion such that strongest peaks are expanded first. TFBM shares the same core principles with the Space Breakdown Method (SBM), a clustering algorithm designed with a similar problem in mind: to differentiate overlapping clusters with distinct densities (Ardelean et al., 2019). During the expansion, BFS uses stopping criteria that ensures ROIs will expand only to the extent of the oscillation packet. First, the TFR point to which it expands must not have been visited before, and it must have a lower power than the current point. This ensures that the expansion is done from the peak on a descending slope. Second, similarly to SBM, the power of the current point must be higher than an expansion bound calculated based on distance and a *dropoff* (see section “3.1.3 ROI expansion and merging” for more details), which essentially stops the expansion algorithm for points of low power situated at some distance from the peak. Each packet center is expanded, iteratively, over all neighboring points that do not meet the stopping criteria. If during the expansions the current ROI finds a point already assigned to another ROI, that point is flagged as a point of “conflict” and a process of “disambiguation” is initiated. The power and *dropoff* of the conflicting peaks, and the distances to the point of conflict are used to decide to which ROI the conflicting point is assigned to. TFBM tends to over-segment the TFR. Therefore, the final step merges the ROIs separated only by small differences/drops in power as described in section “3.1.2 Validation of local maxima.” To summarize, TFBM has three distinct sequential steps: the search for local maxima that become candidate oscillation packets (section “3.1.2 Validation of local maxima”), the expansion of said peaks (section “3.1.3 ROI expansion and merging”) and finally the merging of connected oscillation packets (section “3.1.3 ROI expansion and merging”).

The SBM algorithm is heavily influenced in its performance by the properties of the intensity map, and especially by its local statistics. TFBM inherits the sensitivity to the local statistics of the TFR. For instance, heavily disproportionate time-frequency scales will skew the distance which now needs to operate in the 3D space of time-frequency-power. In order to mitigate this issue, TFBM normalizes the power values such that they are ranged between 0 and 100. The scaling of the time and frequency is not actually performed but it is taken into account while computing the distance on the time-frequency plane with the following modified Euclidean distance:


(1) $$D\,\left(p,q\right)=\sqrt{{\left(s\,c\,a\,l\,e_{t}*\left(p_{t}-q_{t}\right)\right)}^{2}+{\left(s\,c\,a\,l\,e_{f}*\left(p_{f}-q_{f}\right)\right)}^{2}}$$

where *p* and *q* are two points in the time-frequency plane, and *scale*_*x*_ is the scale for a particular dimension, which is computed using the resolution of the TFR in points along dimension *x*:


(2) $$s\,c\,a\,l\,e_{x}=\frac{m\,i\,n\,\_\,p\,o\,i\,n\,t\,s}{p\,o\,i\,n\,t\,s_{x}}*k_{x},\text{with}\,a\,s\,p\,e\,c\,t\,\_\,r\,a\,t\,i\,o=\frac{k_{t}}{k_{f}}$$

where *x* can be time (*t*) or frequency (*f*), and *points*_*x*_ is simply the count of discrete steps along the *x* axis where the TFR is evaluated, and *min_points* is the count of discrete steps of the smallest axis. This equation is used to scale the time-frequency span of the TFR such as to compensate for the large difference between the number of points (*points*_*x*_) in time and frequency. Further scaling can be achieved through the use of the *aspect_ratio* parameter which allows for more resolution on the chosen axis, if not otherwise specified, *k*_*f*_ = 1 and *k*_*t*_ = *aspect_ratio*.

#### 3.1.2 Validation of local maxima

As discussed above, one issue that the algorithm needs to eliminate pertains to the small spurious peaks that appear in noisy regions with very low activity. While inspecting the distribution of power values, we found that usually about 80% of the values fall below 1–5% of the power of the dominant peaks in the TFR (Supplementary Figure 1), which means that most of the dynamic range in the TFR is covered by only a small fraction of the points. In these low-power areas, there are a lot of spurious peaks caused by noise that can be safely removed without any danger of losing legitimate packets. TFBM will automatically compute the threshold such that 80% of the power values from the TFR fall below. In practice, the threshold usually is a small fraction (typically 1–2%) of the maximum power, depending on the signal-to-noise ratio (SNR). However, the threshold is also exposed as a parameter that can be tuned by the experimenter. Importantly, the threshold only affects valid local maxima, the ROI expansion can still use such points. Finally, valid local maxima are not allowed to have another local maximum in their neighborhood. This condition effectively reduces elevated plateaus to one peak and simplifies further processing.

#### 3.1.3 ROI expansion and merging

For TFRs, it is useful to separate ROI expansion and merging in successive steps such that each peak is able to expand the corresponding ROI to the full extent, in order to find and retain its complete time-frequency footprint. Following expansion, the merging process combines packets, whilst both the dominant ROIs and their constitutive sub-packets are retained. This is an important adaptation over SBM because it allows for both coarse-grained and fine-grained investigations, even if it is difficult to decide *a priori* whether an ROI reflects one or multiple underlying processes.

A common feature of SBM and TFBM is the *dropoff* calculation, which has been updated for TFBM to:


(3) D⁢r⁢o⁢p⁢o⁢f⁢f⁢(p)=|T⁢F⁢R⁢(p)−min     (T⁢F⁢R⁢(n))|,f⁢o⁢r⁢n∈N⁢(p)

where *p* is the point in the time-frequency plane at which the *dropoff* is evaluated, *N(p)* is the neighborhood of point *p*, *TFR(p)* is the value of the TFR at point *p*, and *n* iterates through the neighborhood.

In SBM, the *dropoff* of the cluster center candidate is used throughout the expansion. By contrast TFBM recalculates the *dropoff* around each expansion point such that the borders of oscillation packets are delineated more faithfully. The lower bound of the expansion (Figure 1A, gray squares) is calculated as the *dropoff* of the current point multiplied by the distance between the packet center and said point as shown. The expansion condition is described by Equation 4, where *p* indicates the current point of expansion, *n* its neighbors to which it might expand and *PC* the packet center. Figure 1A shows a simple example of the expansion process for each of the three local maxima found, and the extent of each of the peaks. All conflicting points between the current packet and the others are stored in a list to be used in the subsequent disambiguation step.


(4) $$\begin{array}{c} \left[Dropoff\left(p\right)*Dist\left(p,PC\right)<TFR\left(n\right)\right]and\left[TFR\left(n\right)<TFR\left(p\right)\right],\\ \;\,f\,o\,r\,n\in N\,\left(p\right) \end{array}$$

Similar to SBM, TFBM incorporates a disambiguation step. While in SBM, it was achieved during the expansion of clusters, in TFBM it is done as a subsequent step after the expansion. The purpose of the disambiguation is to determine to which cluster/packet should a point be assigned to in the case that multiple clusters/packets could expand to the same point, named a point of conflict. By separating the disambiguation step from expansion, the algorithm can determine for each conflict point all the packets that could assimilate it and then choose the most appropriate. The peak power divided by the distance to the point of conflict is calculated for each candidate peak. The peak with highest value assimilates the conflicting point. Finally, contours can be calculated and the points of conflict for the merging step are defined as the overlap of contours between two packets.

The merging process iterates through all the packets in increasing order of their peak power value and their corresponding conflicting candidates. Two packets are merged if and only if the difference between their peak power values and the maximum power across the conflict points is below a threshold. Additionally, the packet with the higher power value of its peak assimilates the less prominent one. This threshold is exposed to the user as a parameter, called *merge threshold*. The *merge threshold* can be interpreted as the percentage (power values are normalized in the 0–100 interval) of difference allowed between conflicting peaks and their common maximum conflict point.

For each of the found packets TFBM stores the coordinates of the peak, maximum power, prominence as defined in the topological literature (Helman, 2005), coordinates of conflicting points, the contour of the packet, all the point coordinates that form the ROI, and the parent packet if merged. These characteristics completely define and expose the found packets for further analysis.

To summarize, TFBM is controlled by three parameters: (i) the *threshold* which eliminates spurious noisy peaks in the TFR, (ii) the *aspect ratio* which determines the resolution of the TFR for the calculation of the scaled distance, (iii) the *merge threshold* that determines whether two packets will be merged into one or not. All algorithms were implemented, and all analyses were performed with in-house software libraries developed in C# and Python.

### 3.2 Detection of oscillation packets with TFPF

Time-frequency peak finder (TFPF) is inspired by the concept of topographic prominence (Helman, 2005). It is a simple classical threshold-based algorithm that incorporates the concepts of sub-peaks and ROIs that works surprisingly well. In short, the power range in the TFR, from the maximum up to a threshold, is divided into equally spaced cutoff levels (Figure 1B, thin dotted lines). TFPF goes through all cutoff levels, starting from the highest one, and for each level it creates a mask of the TFR with the values above the cutoff level. As the cutoffs get smaller, peaks will appear in the mask as isolated ROIs. As the cutoff levels descend, the ROIs will become larger and larger until some of the ROIs will merge (Figure 1B thick gray line). When this happens, the largest peak in the intersecting ROIs will take ownership of the ROI and will also swallow the smaller peaks. The smaller peaks will be registered as sub-peaks and will retain their ROIs from the previous cutoff level. In this way TFPF will keep track of the merged sub-peaks and their extents as isolated peaks. The algorithm continues until the cutoff reaches the threshold. Finally, the isolated ROIs will define the detected packets. Similar to TFBM, TFPF characterizes the detected packets by a ROI, one bounding box, the frequency and time (corresponding to the dominant peak), and a list with the sub-peaks. Sub-peaks are characterized in the same fashion as the main one.

The threshold is chosen based on the distribution of power values, following the same procedure described for TFBM. The threshold is perhaps the most important parameter for TFPF because it essentially determines how the footprint of the ROI will look like. A high threshold will disregard the noise and render the bursts more separable but can also miss less prominent features. Conversely, with a low threshold many peaks will be incorporated in the same packet (Figure 1B red and yellow peaks). The number of cutoff levels influences how well defined the ROIs of the sub-peaks are. From our experience, this is not a parameter that influences the results heavily as long as the range of power is sampled densely enough.

### 3.3 Detection of oscillation packets with OEvents

OEvents (Neymotin et al., 2022) is designed to identify and analyze oscillation packets in electrophysiological signals by inspecting TFRs. Originally, OEvents was developed on wavelet-based TFRs using 7 cycle Morlets. However, the algorithm is readily usable on other TFRs as well. At the core of OEvents are adaptive thresholds. It first computes the statistics of each frequency of interest across the whole dataset. Next, a local maximum filter is used to detect peaks in the spectrogram, per frequency. Peaks exceeding 4 times the median are considered as moderate-power to high-power events. The authors defined a local power peak within a 3 × 3 window and then seek the time-frequency bounds around it by expanding the peak as long as the power is above a threshold, which is defined as 50% of the peak value or 4× the median, whichever is lower. Next, packets with overlapping areas greater than 50% of the minimum area of each individual event are merged together. Finally, OEvents computes various packet features including frequency span, time span, peak frequency, and other customizable features. The algorithm has been shown to reliably detect the number of cycles and peak frequency of oscillation events with high accuracy for most frequency bands, and at multiple event durations. Here, we are evaluating OEvents in relation to TFPF and TFBM on multiple data sets and on three TFRs, namely the superlet transform (SLT), the continuous wavelet transform (CWT) and the Short-Time Fourier Transform (STFT).

### 3.4 Synthetic data and detection metric

#### 3.4.1 Synthetic data—the atoms and synthetic background

In order to evaluate the detection algorithms, we will often use Gaussian atoms as a ground truth signal. An atom is, by definition, a sine wave packet with a given number of cycles, multiplied with a Gaussian window, where the Gaussian’s standard deviation is set to 1/6 of the sine packet length. The atom vanishes at both ends (Moca et al., 2021), similarly to realistic packets of oscillations, which rise and fall in a short number of cycles. In addition, for the same number of cycles, atoms have a frequency-dependent duration in the same way neural oscillation bursts are shorter at higher frequencies.

To evaluate the three algorithms, we also include synthetic pink and brown noise as backgrounds in which the Gaussian atoms are introduced. Pink noise was generated using the Voss-McCartney algorithm (Whittle, 2006) with 30 coefficient generators (rows). Brown noise was produced by integrating white noise (Gardiner, 2004).

#### 3.4.2 Ground truth data for evaluation

To evaluate and compare various oscillatory burst detection algorithms, we generated synthetic atoms that were then embedded into distracting backgrounds (real EEG data, pink noise and brown noise). Since in the literature, the gamma band seems particularly difficult to handle by detection algorithms (Tal et al., 2020), we embedded the atoms in backgrounds restricted to the 30 to 100 Hz frequency range.

To control the ratio between the atom and the background, we used the following definition of signal-to-noise ratio (SNR):


(5) $$S\,N\,R=\frac{V\,a\,r\,\left(x\right)}{V\,a\,r\,\left(y\right)}$$

where *x* and *y* are two signals. Please note that for a signal with zero mean (no DC offset), as is the case here, the variance of the signal in time is actually its power. Our choice of SNR is motivated by the fact that the detection algorithms operate on TFRs, which in most cases show power. In this form, the SNR is linear in the power domain, and it is best suited to compare the detection algorithms.

For the background, we used one channel EEG data (PZ), and pink and brown noise filtered to the band of interest (30–100 Hz) with bidirectional IIR filter (3rd order Butterworth). The variance of the filtered signal will essentially be the power within the band of interest. To scale an atom, *a*, with a scaling factor, *k*, to match a desired SNR with respect to the background, *b*, the relation is:


(6) $$S\,N\,R=\frac{V\,a\,r\,\left(k\cdot a\right)}{V\,a\,r\,\left(b\right)}$$

Then the scaling factor is:


(7) $$k=\sqrt{S\,N\,R}\,\frac{S\,t\,d\,e\,v\,\left(b\right)}{S\,t\,d\,e\,v\,\left(a\right)}$$

#### 3.4.3 Detection metrics

To compare the algorithms, we need an error function, able to compare the ROIs of packets detected by the algorithm against the ground truth, the atom’s known ROI. Or, in other words, we need a measure of how well two ROIs match. To that end, we define the match measure, *m*, as the ratio between the ROIs’ intersection and reunion (Figure 1C left):


(8) $$m\,\left(A,B\right)=\frac{A\cap B}{A\cup B}$$

where *A* and *B* are the two ROIs. The match *m* is 1 if the intersection and reunion are identical, that is if the two ROIs completely overlap, otherwise *m* has a sub unitary value. If there is no intersection, then *m* becomes 0. The matching error can then be defined based on *m*:


(9) e⁢(A,B)=1-m⁢(A,B)

Consider an atom embedded in the background noise. The detection algorithms will identify a series of packets in the TFR out of which only one, if any, will have the best match with the atom’s true ROI (Figure 1C right). The best matching packet will produce the lowest error (Equation 9), when its ROI, *B*, is compared to the atoms known ROI, *A*. If none of the detected packets match the inserted atom, then we consider that the atom was not detected in that trial. The ground truth ROI *A* and its bounding box are defined based on the atom’s noise-free TFR. The area with power above 20% of its peak power forms the ROI and defines the bounding box. These surface matching measures are readily transferrable to bounding boxes.

### 3.5 Time-frequency representations

In the following, we will evaluate TFBM, TFPF, and OEvents using the SLT, CWT and STFT. All particular analysis parameters for all figures are displayed in Table 1.

**TABLE 1:** Analysis parameters.

	Analysis and corresponding figure	Figures 2A,C,D	Figure 2B	Figure 3	Figure 4 and Supplementary Figure 3	Supplementary Figure 2	Supplementary Figure 4
Algorithm	Parameter						
TFBM	Threshold (percentile)	90%	92%	90%	90%	92%	80%
	Merge threshold	35%	35%	15%	15%	35%	15%
	Aspect Ratio	1	1	1	1	1	1
TFPF	Threshold (percentile)	90%	92%	90%	90%	92%	80%
	Cutoff levels	30	30	30	30	30	30
OEvents	Threshold (×median)	4	4	4	4	4	4
SLT	Base cycles, *c*_1_	3	3	3	3	3	3
	Order, *o*	10:10	5:10	10:10	10:10	5:10	10:10
CWT	Cycles	–	–	–	7	7	7
STFT	Window size	–	–	–	250 ms	250 ms	250 ms
	Step size	–	–	–	1 ms	1 ms	1 ms
	Frequency resolution	–	–	–	4 bins/Hz	4 bins/Hz	4 bins/Hz
	Window type	–	–	–	Blackman	Blackman	Blackman

For each algorithm and analysis, all parameters are listed with reference to the figure it pertains to.

Here, we used the SLT transform (Moca et al., 2021) with base cycles *c*_1_ = 3 and order *o* = 10, which provides a non-diluting TFR with good time-frequency resolution. For illustrative purposes, the adaptive SLT with order *o* = 5:10 was also used in order to better resolve high-frequency oscillations. For the CWT we used Morlet wavelet with 7 base cycles, as used originally in the OEvents paper (Neymotin et al., 2022). For the STFT we used a Blackman window of 250 ms slid over the data with a step of 1 ms. Zero padding was used in order to increase the frequency resolution to 4 bins/Hz. The SLT and CWT were computed using the same frequency resolution.

## 4 Results

### 4.1 Structure of identified oscillations

**FIGURE 2:**
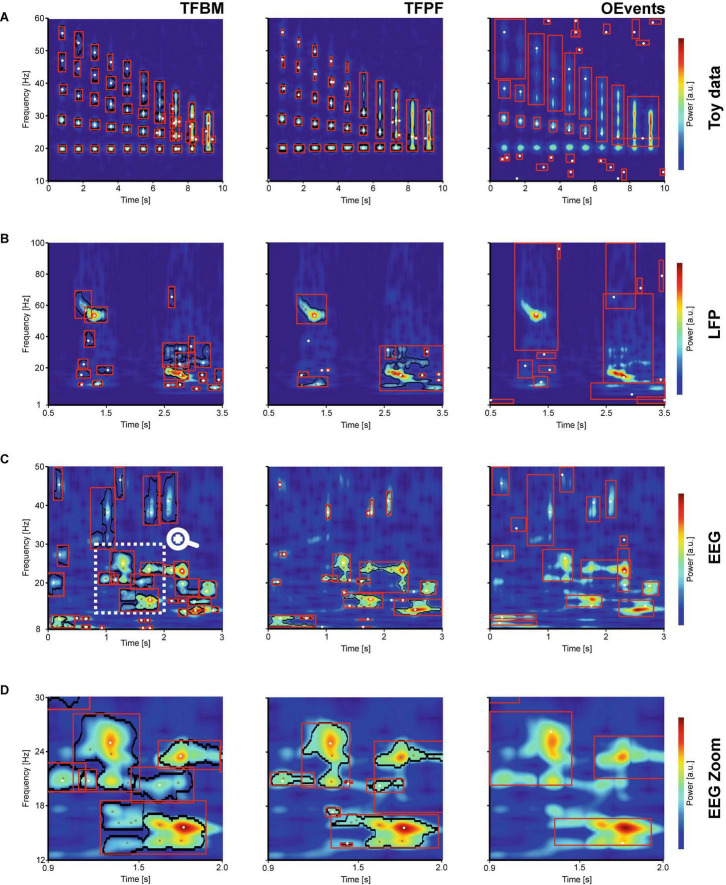
Single trial comparison. The figure compares the segmentation of TFBM (left), TFPF (center), and OEvents (right) on several single-trial TFRs computed with superlets. The ROI boundaries of detected packets are shown in black and the bounding boxes in red. Local maxima are marked with white dots and sub-peaks with gray. **(A)** A series of atoms in the gamma range (20–55 Hz) with increase proximity in frequency are embedded in uniform noise. **(B)** A wide frequency range (0–100 Hz) of a single trial LFP recording from mouse visual cortex, stimulated with a drifting oriented bar. **(C)** Single trial electroencephalogram (EEG) with rich spectrum covering the alpha, beta, and low gamma frequency band. The marked area in **(C)** is zoomed-in in **(D)**.

We first compared the structure of identified packets between the three algorithms (Figure 2). In all tests, if not otherwise specified, the data was analyzed with the adaptive superlet transform (SLT) and all algorithms were applied to the same time-frequency representation (TFR). Even if OEvents was initially used in conjunction with a wavelet TFR, here we wanted to factor out from evaluations the differences between TFRs. In principle all three algorithms can be used in conjunction with any TFR, and all can potentially benefit from the sharper spectral representation of the superlets. However, for the sake of completeness we also evaluate their performance on the Short-Time Fourier Transform (STFT) and the continuous wavelet transform (CWT). All parameters used for the analyses are summarized in Table 1.

With the first test we wanted to probe the capacity of the methods to delineate different processes that have a close proximity in the frequency space. To that end, we generated groups of Gaussian atoms (20 cycles) equally spaced in time but progressively closer in the frequency space (Figure 2A). The atoms with amplitude 1 were embedded in a uniform random noise with double the amplitude. The signals were analyzed with the SLT (base cycles *c*_1_ = 3, order *o* = 10:10) and all algorithms were applied on the same TFR. In the following paragraphs, we focus on: (i) the ability to detect existing atoms, without introducing spurious nonexistent oscillations, (ii) over-segmentation, and (iii) the ability to separate distinct atoms.

Time-frequency breakdown method (TFBM) and time-frequency peak finder (TFPF) were both able to identify all the atoms, while OEvents struggled to detect the atoms due to its per-frequency thresholding logic that requires power to be 4 times above the median for a peak to be detected (Neymotin et al., 2022). In this particular test, due to the amplitude of the atoms and the noise, the OEvents median is not able to separate clear oscillations from the background. At low frequency (20 Hz), where atoms introduce a lot of power, the median is too high and clear bursts are missed. At higher frequencies, where shorter atoms introduce less power, the normalization makes even tiny levels of power seem significant and many atoms are clumped together under the same bounding box, as a single event. Symptom of the same problem, a lot of non-existing events are detected as oscillations in areas without atoms. Overall, OEvents misses and merges the most while detecting spurious oscillations in areas with noise. We have also run the same OEvents analysis, but the median used for normalization was computed over the entire TFR and the same value was used on all frequencies. The reasoning was that, when only short stretches of signals are available, the median over the entire TFR is more representative of the data. This is especially the case with TFRs, such as superlets, where 1/*f* dilution is less pronounced. Indeed, using one median across the entire TFR was a better normalization for this particular case, and OEvents performed significantly better, but still merged more atoms together than TFBM and TFPF (data not shown).

A closer look at TFBM shows that some of the atoms were subdivided or sometimes broken into distinct subparts (adjacent red bounding boxes in Figure 2A left pane). This is in part due to the parameter choice (see Table 1), which in this case favors a finer segmentation designed to separate as much as possible the overlapping packets. Indeed, TFBM was able to separate packets slightly better than TFPF, but at the cost of over-segmentation. TFPF on the other hand, is a simple threshold-based method, which works well when the packets are quite clear, like in this example. It performs almost the same over segmentations as TFBM, but the over-segmented satellite peaks are very small and less clearly apparent. As compared to TFBM, it is more difficult to tune its sole parameter, the threshold, to detect the faint packets and at the same time to separate well the overlapping packets. Notably, with these parameters, none of the packets are missed by TFBM or TFPF, albeit with some over-segmentations and spurious merges where atoms are close in time and frequency (above the 7s time mark). These “spurious” packets are faint parts of already existing packets and could be eliminated by thresholding the representation at slightly higher power values.

In Figure 2B, we show an example of single-trial local field potentials (LFP) recorded from mouse visual cortex (V1) during a receptive field mapping trial, where a moving bar traverses horizontally the receptive field. The adaptive SLT used here was optimized (Table 1) to cover a wide frequency range (1–100 Hz). It reveals a wide array of frequencies with well-defined bursts of activity, such as the gamma burst at 55 Hz at around 1s and the beta burst that starts at 2.5s. Both have a complex shape with decreasing frequency and power modulations that appear as sub-peaks. In addition, there are several small and fainter oscillations that enrich the landscape. TFBM and TFPF are able to delineate (black border) the most important packets of oscillations. Between TFBM and TFPF, TFBM does a better job at isolating both large and small packets, some that are actually missed by TFPF. Take for instance the 35 Hz burst that occurs immediately after the 1s mark. Although faint, it is clearly separable from the background and has a series of easily identifiable sub-peak. All these aspects are correctly revealed only by TFBM. After 2.5s the beta-gamma activity is also better segmented by TFBM which detects a larger number of structures that, at least subjectively, should be separated. TFPF also nicely delineates these structures, but it performs more aggressive merging. Note, however, that the beta (14 Hz) and gamma (55 Hz) packets at 1s are over-segmented by TFBM but nicely isolated by TFPF. OEvents behaves similar to the previous example, over-merging bursts across the entire spectrum. Important to note is that both TFBM and TFPF do a good job at carving out oscillation packets that are clearly delineated, with sub-peaks correctly placed over prominent sub-peaks. TFBM and TFPF present an unprecedented level of detail with respect to the complexity of the identified structure and the level of description. To identify such structures, we argue that it is important to employ the appropriate TFR. In Supplementary Figure 2 we compare the algorithms on three different TFRs namely the superlet, CWT, and STFT. The same data as shown in Figure 2B was used. The comparison shows how the crisp details in the superlets allow the algorithms to overall better separate the structures.

The SLT shows the same level of detail in human EEG data (Figure 2C). Here, OEvents performs better than in the previous two cases and identifies the most prominent features of the TFR. The OEvents segmentation is in general greedier and merges larger areas in a coarser account of the structures, as compared to TFBM and TFPF. It also misses some of the packets with low power such as the 20 Hz burst starting at 0s or the brief alpha activity around 1.5–2s. TFPF also misses or barely finds packets in higher frequencies. By contrast TFBM is the algorithm that best delineates the structures, although it is merging some of the faint packets. For instance, it merges together, like OEvents, the 30–40 Hz structures around the 1s mark. TFBM identifies more structures than TFPF and OEvents. The alpha activity and the beta (20 Hz) activity around 1.6 and 2.5s are correctly delineated only by TFBM. Overall, the TFBM segmentation is superior to TFPF but perhaps overly greedy in faint areas, while OEvents captures only a rough structure of the oscillations.

Take, for instance, the rich region between 0.9–2.05s in the 12–30 Hz range which is zoomed in Figure 2D. In this area, the oscillations are clear and TFBM’s segmentation borders follow the edges of the oscillations most accurately (Figure 2D left). TFPF also finds the contours nicely, but it suffers from the fixed threshold and poorly delineates smaller peaks, such as the low beta burst just before the 1.5s mark. By comparison, OEvents’ bounding boxes (Figure 2D right) cannot match the level of details put forth by the contours of TFBM or TFPF.

### 4.2 Comparison between methods

As we have seen from the examples in Figures 2B–D, single trial neural oscillations are usually embedded in rich landscapes, with background activity and noise. In the following, we tested how well the three methods are able to recover known oscillations buried in realistic backgrounds. To this end, we generated 200 atoms with a duration of 10 cycles and frequencies ranging from 35 to 95 Hz. Each atom is assigned a random frequency and insertion time point after which is inserted into a background instance. Following the procedure described in section “3.4.2 Ground truth data for evaluation,” we calibrated all atoms’ amplitude to obtain increasingly higher SNR levels (0.1, 0.25, 0.5, 1, and 2) and we then embedded them in the data, such that the same configuration of the atoms’ frequency, time, and trial were repeated for each SNR. In summary, 1,000 tests have been performed using 200 atoms at five SNR levels.

**FIGURE 3:**
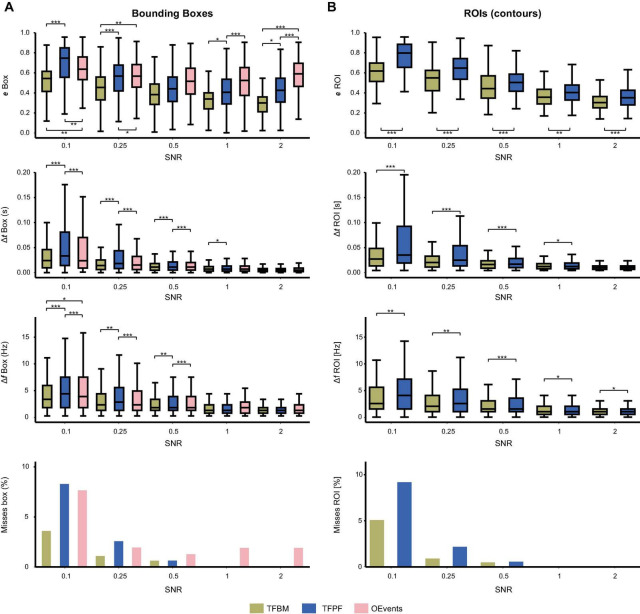
Detection comparison between the three algorithms as a function of SNR. In **(A)** the match between the ground truth (atom) and the detected packets is evaluated using the matching error of rectangular bounding boxes. The top panel shows the bounding-box detected errors with better detection as SNR is increased. Time and frequency errors are shown on the second and third panels, respectively. Finally, the percentage of missed atoms is shown on the bottom. For TFBM and TFPF **(B)** shows the same data as in **(A)** but evaluated using the fine-grained ROIs (contours) instead of the bounding-boxes. Stars indicate significance levels.

First, we embedded the atoms in EEG data to test the detection in a context as realistic as possible. To that end, for each atom one trial is chosen randomly from the 84 available EEG trials. Figure 3A shows the detection measurement statistics when all three algorithms were evaluated based on the rectangular bounding boxes of the detected packets. As described in section “3.4.3 Detection metrics,” a known atom was considered detected if its bounding box intersected with at least one of the packets found by the detection algorithm. The best matching packet is then considered for error measurement against the ground truth atom. Here, we measure three errors: the bounding box match error, the time match error, and the frequency match errors. The latter two are just the differences between the location in time and frequency of the atom and that of the highest peak in the bounding box. However, we also measure the percentage of missed atoms (no detected packet has any overlap with the atom).

At all SNRs, TFBM is able to locate the atoms with significantly lower error than the other two algorithms (Figure 3A, top). This is perhaps due to the fact that TFBM is best at capturing packets at low SNRs, where it has two times fewer misses than TFPF and OEvents. The latter two fail to find about 7% of the atoms at SNR 0.1 (Figure 3A, bottom). Overall, TFBM shows lower bounding box errors than TFPF and OEvents at all SNR levels and also the smallest proportion of missed packets. From SNR = 1 onward, none of the packets are missed by TFBM and TFPF. At low SNRs (≤0.25) TFPF’s performance is slightly lower than OEvents, but at SNRs ≥1 TFPF has lower bounding box errors and no misses.

Next, we evaluate the time and frequency errors (Figure 3A, second and third panel, respectively). Time and frequency errors can be computed only if the atom is detected. Consequently, atoms that were not detected did not contribute to the time and frequency error statistics. At SNR = 0.1, TFPF and OEvents lose twice more atoms than TFBM (Figure 3A, bottom). This favors their time/frequency distributions since the missed, hardest to detect packets, do not contribute to their error distributions but are registered by TFBM. However, even in this case time and frequency errors are smaller for TFBM. Overall, errors decrease as a function of SNR, and starting with SNR = 1 and above the time and frequency errors are extremely low and the three algorithms performed with the same accuracy in all these tests. Nevertheless, OEvents still misses a small fraction of the atoms while and TFPF and TFBM miss none. For SNR ≤ 0.5 TFPF had a harder time in determining the correct timing and frequency of the atoms than TFBM and OEvents. All significance levels were assessed with a paired *t*-test, assuming unequal variances, with Bonferroni correction for multiple comparisons, where applicable (Figure 3).

The same procedure is applied for measurements shown in Figure 3B, but rather than using the rectangular bounding boxes, the evaluations are performed on the detected ROIs (surface bounded by the detected packets’ contour). Only TFPF and TFBM were compared, because OEvents finds only the bounding boxes. In terms of contours matching TFBM is significantly better than TFPF across all SNRs. At SNR = 0.1, TFBM only misses about 5% of the packets as opposed to TFPF, which fails to find about 9% of the packets. From SNR 1 and above, when the atoms stand out from the background, the TFBM and TFPF algorithms have virtually the same performance in terms of frequency and time errors, and no packet is missed.

In previous evaluations we have used SLT as the TFR of choice and we embedded atoms in background EEG. These raises two questions: How would the algorithms perform on other TFRs and what is the influence of the background of choice? Therefore, in the next evaluations we aim to answer these two questions.

**FIGURE 4:**
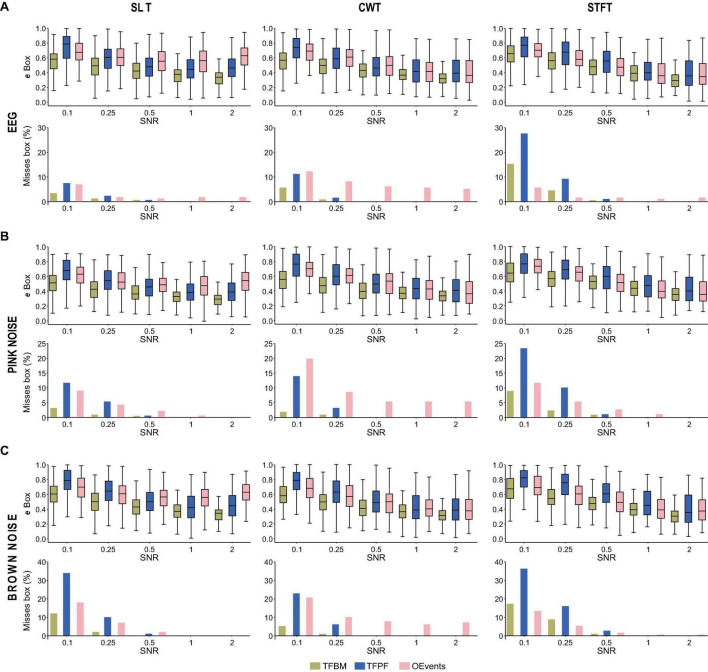
Effects of background type and time-frequency representations on detection. **(A)** Shows the detection performance of atoms embedded in electroencephalogram (EEG) background. The box match error (upper panes) and the percentage of missed packets (bottom panes) are shown for three TFRs: SLT on the left, CWT in center and STFT on the right. For easy comparison, left panes in A, recapitulate top and bottom panes in Figure 3A. In **(B,C)** the same evaluation is shown for backgrounds of pink and brown noise, respectively.

In a second set of comparisons, we ran the detection algorithm on the same atoms embedded in EEG, but using also CWT and STFT, the two most established TFRs. Figure 4A illustrates the comparative performance of TFBM, TFPF and OEvents on the three TFRs generated by SLT, CWT, and STFT. As expected, for all representations, the algorithms perform better for increased SNR. TFBM has the smallest box errors in most situations and performs best in combination with SLT. It also fares better than TFPF and OEvents for CWT. Overall, TFBM and TFPF profit most from the sharper (Supplementary Figure 3) superlet. By contrast, OEvents seem to fare comparably well on SLT and STFT, with most misses on CWT (see also Supplementary Figure 4).

Time-frequency peak finder (TFPF) and TFBM have problems on STFT at SNR ≤ 0.25, where they miss more atoms than OEvents. The fact that both TFPF and TFBM have a problem on STFT at low SNR could be caused by the thresholding operation that eliminates small power values. The thresholds were established on the SLT representations, while the STFT has different spectral characteristics. To test this hypothesis, we lowered the power threshold such that more of the low part of the TFRs contributes to packet detection (Supplementary Figure 4A). Indeed, with a lower threshold (80%) the detection performances of TFBM and TFPF greatly improve and they always surpass OEvents. With this lower threshold that favors the detection of atoms, TFBM only misses atoms on STFT and less than 2% at SNR = 0.1. TFPF also barely loses packets on SLT and CWT. These results point to SLT as being the TFR of choice for TFBM and TFPF.

Third, because EEG might not be the most suitable background in all situations, we also tested atom detection with pink noise (Figure 4B) and brown noise (Figure 4C) backgrounds generated according to the procedures described in section “3.4.1 Synthetic data—the atoms and synthetic background.” Similarly, to the case of EEG background, each atom is embedded in a different instantiation (trial) of the noise. The same procedure for embedding (including filtering) was performed, as described in section “3.4.3 Detection metrics.” In terms of performance the algorithms perform similarly on background noise and EEG. With one exception (brown noise background with STFT) TFBM performs best across the board. OEvents and TFPF are competing at small SNRs (≤0.25) while at SNR ≥ 0.5 TFPF has fewer overall misses than OEevents (except at brown noise with STFT and SNR = 0.5). Lowering the power threshold dramatically improves packet detection for TFBM and TFPF also in the case of brown and pink backgrounds (Supplementary Figures 4B, C). With TFPF and TFBM missing far less packets as compared to OEvents (except TFPF on brown noise with STFT at SNR = 0.1), even though the box error was usually larger for TFPF than for OEvents. As in the case of EEG background, TFPF and TFBM usually performed better on SLT and worse on STFT, with OEvents faring worse on CWT (Figures 4B, C and Supplementary Figures 4B, C).

## 5 Discussion

Detection of oscillation transients in neural data can be a challenging task due to their 1/*f* nature and the rich time-frequency landscape they populate. The packet detection methods introduced here can work, in principle, with any time-frequency representation (TFR), estimated using Fourier analysis, wavelets, Wigner-Ville distributions, etc. We argue that the properties of the TFRs have a critical impact on the detection performance of any methods that rely on them for finding oscillation transients. Therefore, instead of focusing on tuning packet detection algorithms to cope with the 1/*f* nature of neural signals, for TFR-based methods, one should instead focus on improving the TFR itself, or applying TFR correction techniques, such as the pseudo Z-score baselining (Ciuparu and Mureşan, 2016). When applicable, the baselining technique enables the removal of the 1/*f* trend from the TFR, irrespective of the technique used to estimate the latter. Nevertheless, the way the TFR concentrates the representation of an oscillation transient, simultaneously in time-frequency, still remains an important factor, even if baselining is applied. The shape, amplitude, and time-frequency span of an oscillation event extracted by a detection algorithm depends ultimately on the time-frequency estimation method, i.e., the TFR. We discuss these issues next.

### 5.1 Importance of TFRs

The development of capable TFRs is a very active field of research in signal processing (Pachori and Sircar, 2007; Boashash, 2015; Yu et al., 2019; Peng et al., 2020; Li et al., 2022). TFRs are estimators—the true time-frequency representation of a signal is usually not known. For obvious reasons, methods that rely on TFRs to detect oscillation transients depend dramatically on the ability of such TFRs to correctly estimate the presence of the transients. The two most widely used TFRs are built using the Short-Time Fourier Transform (STFT), to calculate spectrograms, and the continuous wavelet transform (CWT), to calculate scalograms. Both have serious issues. The spectrogram uses a fixed sliding window to estimate the evolution of power in time. The window needs to be large enough to capture the lowest frequency in the representation. As a result, higher frequency bursts, which are shorter in time, will fill a smaller proportion of the window and appear “diluted” in the representation (Moca et al., 2021). On the other hand, the traditional scalogram uses wavelets with energy preserving normalization. This also suffers from power dilution [see Supplementary Information in Moca et al. (2021)]. Power dilution of both representations exacerbates the 1/*f* problem by even further attenuating spectral components in the higher frequency range. A solution around the dilution problem for wavelet-based techniques is a wavelet normalization that conserves the modulus integral (Mallat and Zhong, 1992; Liu et al., 2007; Lilly, 2017)—by default implemented in Matlab. In such representations, the peak power density of an oscillation burst with a finite number of cycles will be the same at any frequency. However, this leads to another issue, called redundancy: the total energy recovered from the representation is larger than the actual energy of the signal. Nevertheless, these representations are very useful for the detection of oscillation packets (Moca et al., 2021).

Not only dilution or the 1/*f* nature of brain signals poses challenges. Perhaps the most formidable one is the Heisenberg-Gabor uncertainty principle, or the Gabor limit (Gabor, 1946; Heisenberg, 1985), according to which one cannot perfectly localize a signal in both time and frequency simultaneously. This introduces a resolution limit, which can be theoretically achieved by the family of Wigner-Ville distributions (WVD) (Boashash, 2015). Unfortunately, the price to pay to achieve such resolution is that WVDs exhibit cross-terms (artefactual components in the spectrum), which render them unusable for data with a rich spectral landscape, such as brain signals (Moca et al., 2021). The more traditional techniques are usually not even coming close to the resolution limit displaying leakage of power across frequencies and/or time-smearing. Therefore, even well-defined oscillation packets may be difficult to detect in such representations, especially when close-by components exist, whose representation is mixed with that of the target packets. Therefore, methods are needed that can achieve good or near-optimal power concentration, yielding good time-frequency resolution.

One such method is the SLT, which combines multiple wavelet representations geometrically, to achieve time-frequency super-resolution: the SLT has a better resolution in the time-frequency space than any of the individual wavelet representations from its set (Moca et al., 2021). Another advantage of the SLT is that it uses the modulus integral wavelet normalization, such that the representation does not suffer from dilution issues, alleviating the 1/*f* problem. Here, we have shown that using superlets can enable packet detection methods to isolate target oscillations with remarkable precision. In general, any detection algorithm that uses TFRs could benefit from the improved time-frequency landscape provided by superlets.

### 5.2 Detection methods

Detection methods based on TFRs differ in terms of the particular algorithms used to detect and isolate peaks in the representation. Aside from the normalization to compensate for 1/*f* phenomena, most algorithms use some kind of local peak seeking (Tal et al., 2020). Then, rectangular bounding boxes are typically constructed to delineate the estimated extent of the packet at the location of the peak (Neymotin et al., 2022), but see Waldman et al. (2018). However, packets can frequently exhibit less canonical shapes in the TFR, like bursts with increasing or decreasing frequency, ripples on spikes (Waldman et al., 2018), etc. It would therefore be ideal to devise methods that can perform detection in such a way that the precise contour of the packet can be extracted.

Here, we developed two methods that are able to estimate the presence of oscillation packets in complex TFRs of brain signals, while also extracting the contour of these packets. The geographical approach of time-frequency peak finder (TFPF) bears some similarities with the algorithms used by Waldman and colleagues (Waldman et al., 2018) to identify RonS high-frequency oscillations (HFOs). Although the RonS detection algorithm employs contours, these are mainly used to detect whether the packet is limited to the ripple band or if it is a broad-band artifact. In addition, similar to other packet detection algorithms, the RonS detector is specifically designed for one type of activity, the RonS HFOs, and is not readily transferable to other frequency bands or to packets with different morphologies. In our case, time-frequency breakdown method (TFBM) and time-frequency peak finder (TFPF) were developed with the clear target in mind of identifying the precise contour of various packet morphologies in TFRs, rather than just their rectangular bounding box. In addition, it would be desirable to have techniques that are able to determine the hierarchical relationship between different, nearby TFR peaks. We have shown that both TFBM and TFPF can successfully meet these two requirements.

When compared to the other techniques, TFBM is more complex but appears to have a better performance, especially on difficult cases. Indeed, TFBM has lower errors than both TFPF and OEvents (Tal et al., 2020), irrespective of the type of TFR or the statistics of the background (EEG, pink noise, or brown noise). With respect to the misses of packets, TFBM seems best suited to operate on the SLT representation, where it clearly outperforms all the other methods and obtains the best results overall. The OEvents fares best on the STFT, sometimes suffering from less misses than the TFBM at low SNR on this representation. This is likely because, on the STFT, OEvents takes advantage of the frequency-wise normalization, which has not been applied on the TFR of the TFBM—although a pseudo Z-scoring (Ciuparu and Mureşan, 2016) could be applied and may bring TFBM in advantage. Regarding the type of TFR, at low signal-to-noise ratio, the SLT generally provides the lowest detection error irrespective of the type of detection technique used (Supplementary Figure 3), indicating the advantage this representation brings in the case of complex TFR landscapes, cluttered with noise. From the extensive testing performed here, the best results are obtained by the combination of TFBM-SLT, across all types of data, with strong performance boosts at low signal-to-noise ratios.

Beyond performance, TFBM and TFPF have an advantage over previous methods because they can delineate the fine contour of the detected oscillation events. Previous methods, with the exception of RonS detection algorithms (Waldman et al., 2018), can only extract rectangular bounding boxes of oscillation packets. While the RonS algorithm also employs contours, the algorithm is constructed for a specific type of HFO, the RonS, and is not readily extensible to other type of packets (oscillations or not) or to other frequency bands. Importantly, the ability of the novel methods introduced here to construct hierarchical relationships between components of the TFR can be exploited for unprecedentedly detailed analysis of the underlying bursting processes.

Finally, while there seems to be no perfect methods for detection of oscillation packets, in general more complex ones, like the TFBM, seem to fare better. The price to pay, of course, is increased complexity and more parameters, which may need to be tuned by the user. OEvents, for example, features a fixed threshold (4 * the median threshold), and perhaps this could be tuned in order to obtain better performance on certain types of representations / signals. Similarly, TFBM features a single parameter that needs to be manually provided, the rest are optional and are calculated automatically. Nevertheless, these optional parameters are also exposed and can be set manually. In all cases, however, on real neural data, choosing an optimal parameter set is a tedious undertaking, because the ground truth is not known.

### 5.3 Single-trial analysis versus averaged TFRs

It is worth pointing out that even isolated bursts can be misinterpreted as sustained oscillations in averaged TFRs. This is a confound ultimately rooted in the high variability of neuronal responses, frequently overwhelming the effects of interest. For example, an assumption that is often made is that stimulation induces effects that are small relative to background activity, such that, for a reliable detection of these effects, multiple realizations (trials) under the same condition are required. However, the usual averaging of the spectra does come with confounds. In power spectra both induced and evoked effects sum up, including those oscillation bursts not time locked to the stimulus. The first consequence is that induced, non-time-locked, activity gets smeared out and diluted by the background. Second, if oscillation bursts at a fixed frequency are spread out in time, the resulting averaged TFR has a smeared-out appearance that may be confounded with a sustained oscillation, even if single trials do not contain sustained rhythms. We therefore argue that the true nature of oscillation packets can only be revealed by performing single trial analyses.

## 6 Conclusion

Here, we have introduced two robust techniques for detecting, isolating, and quantifying oscillation packets of any shape and expressed anywhere in TFRs of neural signals. They are able to precisely isolate the shape of the time-frequency components in the TFR and to determine hierarchical relationships between peaks that represent oscillation packets. In addition, we also demonstrated the usefulness of super-resolution TFRs for the detection of oscillation packets. In particular, the recently developed superlet transform appears to fare well on neural data. Such powerful tools to characterize oscillation bursts open the way for quantitative analysis of the properties of transient oscillations in brain signals. For example, one hypothesis that could be explored is that oscillation events are manifestations of scale-free processes operating in cortical circuits (Grosu et al., 2022), which give rise to periodic transients that are self-similar (fractal) across a wide range of temporal scales. The power of tools for detection of oscillation packets is yet to be revealed by complex future studies of transient oscillation processes.

## Data availability statement

The raw data supporting the conclusions of this article will be made available by the corresponding authors, upon reasonable request. Free source code with a Python implementation of the methods can be found at: https://github.com/TransylvanianInstituteOfNeuroscience/OscillationDetection.

## Ethics statement

All animal procedures were approved by the Local Ethics Committee (3/CE/02.11.2018) and National Sanitary and Veterinary Authority (147/04.12.2018) and conducted in accordance with the ethical guidelines of the European Communities Council Directive 2010/63/EU and Romanian Law 43/2014. Experiments involving human participants were carried out in compliance with Directive (EU) 2016/680 and Romanian Law 190/2018 and were reviewed and authorized by the local ethics committee (1/CE/08.01.2018). All participants involved in the study provided their written informed consent.

## Author contributions

E-RA and HB developed the algorithms, implemented, tested, and performed the comparisons between the methods. HB generated the toy data. A-MI, HB, RM, and VM recorded the test data. VM and RM designed the tests and derived analytical formulations. VM, RM, E-RA, and A-MI wrote the manuscript. All authors contributed to the article and approved the submitted version.
