# Erratum: Sharp detection of oscillation packets in rich time-frequency representations of neural signals

**DOI:** 10.3389/fnhum.2024.1397042

**Published:** 2024-03-18

**Authors:** Frontiers Production Office

**Affiliations:** Frontiers Media SA, Lausanne, Switzerland

**Keywords:** neural oscillations, burst, detection, quantification, time-frequency spectrum, superlets

Due to a production error, information regarding code availability was not included. The **Data availability statement** has now been corrected to state:

“The raw data supporting the conclusions of this article will be made available by the corresponding authors, upon reasonable request. Free source code with a Python implementation of the methods can be found at: https://github.com/TransylvanianInstituteOfNeuroscience/OscillationDetection.”

The publisher apologizes for this mistake. The original article has been updated.

