# Supplementary material for: Sharp detection of oscillation packets in rich time-frequency representations of neural signals
Source: Front Hum Neurosci. 2023 Dec 7;17:1112415. doi: 10.3389/fnhum.2023.1112415 (PMC10748759; doi:10.3389/fnhum.2023.1112415)
Supplement: Supplementary file 1 [file Data_Sheet_1.PDF]

Supplementary Material

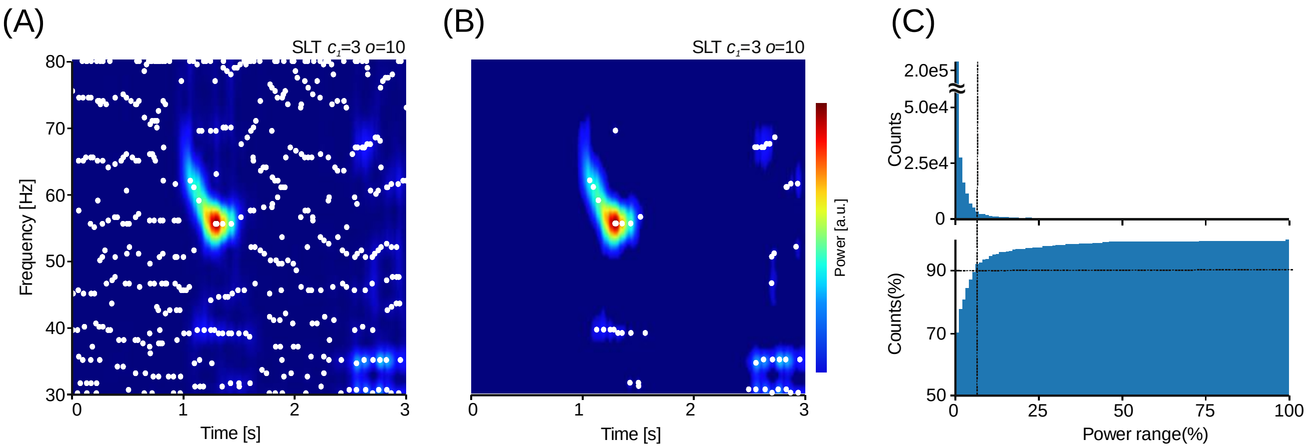

Supplementary Figure 1. Thresholding and rejection of the spurious low amplitude peaks. All local maxima found in a superlet TFR of a single-trial mouse LFP are shown in (A), while (B) shows only the local maxima above the threshold determined according to (C). In (B) all power values below the threshold have been set to 0 in order to emphasize the low threshold which allows the TFR to retains its dynamic range. (C) The top panel shows the highly skewed distribution of the power values in (A). The cumulative distribution (bottom panel) is used to set the threshold to cover 90% of all power values, which translate into less than 10% of the maximum power. For easy interpretation, the power values in the TFR have been scaled to the interval 0-100.

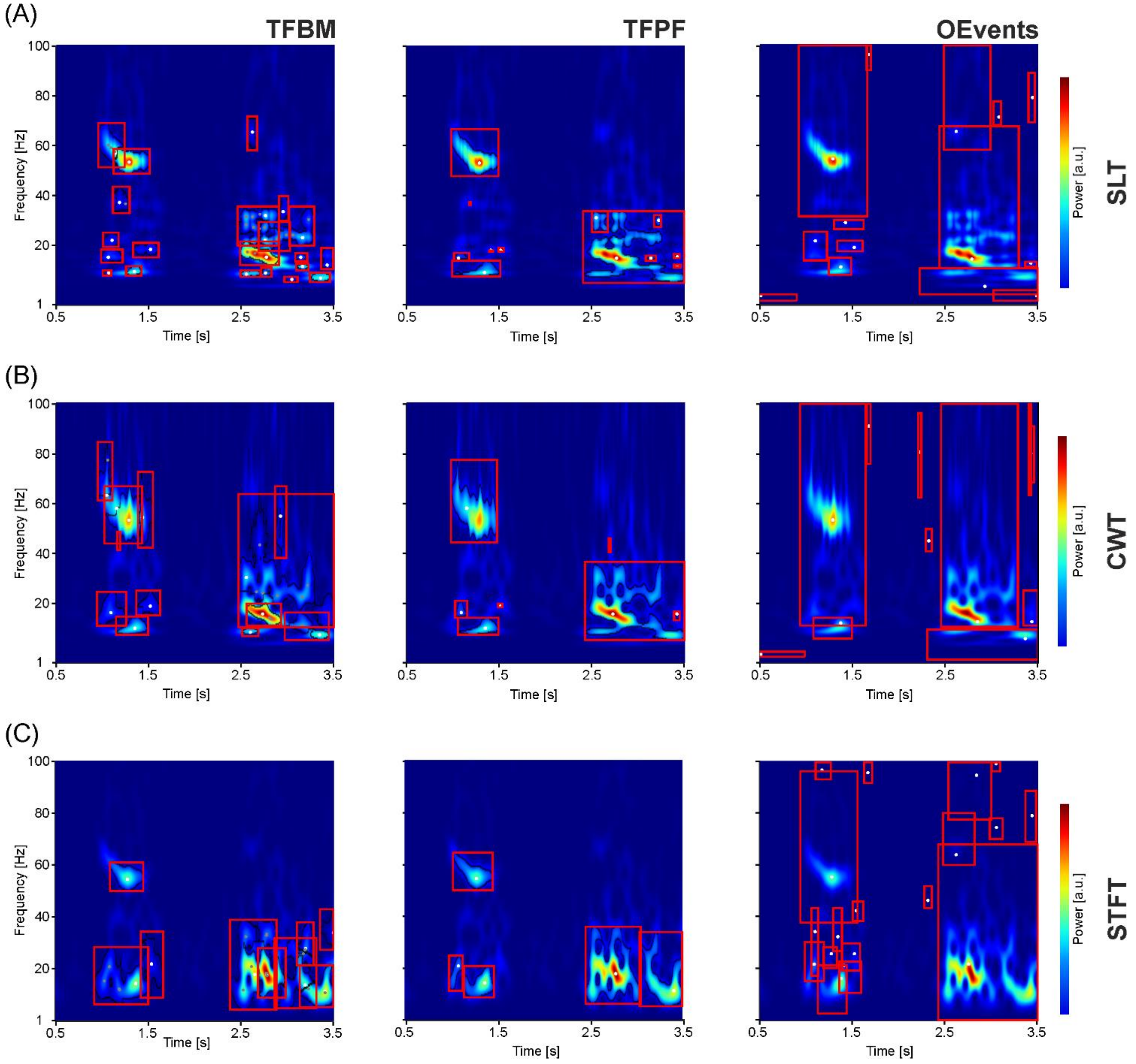

Supplementary Figure 2. TFR influence over packets detection. (A) The segmentation over one LFP trial between TFBM, TFPF, and OEvents, respectively. For clarity, this pane recapitulates Figure 2B as it shows a different comparison, this time against other time-frequency representations. (B) and (C) have the same format as (A) but using the CWT and STFT time-frequency representations, respectively.

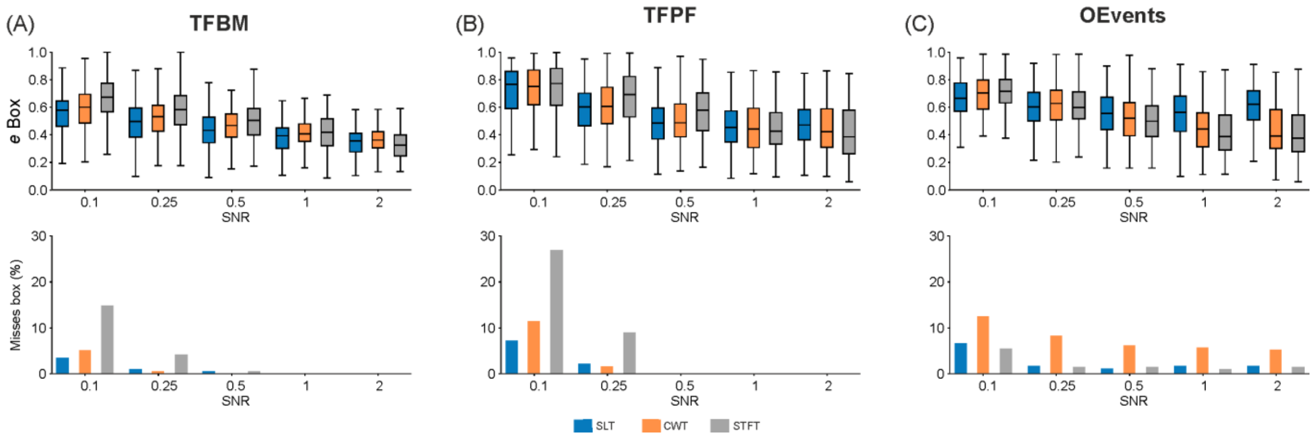

Supplementary Figure 3. Atom detection performance in SLT, CWT, and STFT time-frequency representations. (A) TFBM detection performance (bounding box error) as a function of SNR for the three TFRs (SLT, CWT, STFT) is shown in the upper pane, while percentages of missed atoms are shown on the lower pane. (B) Same as in A, but for the TFPF algorithm. Here, TFPF has the most misses on low SNR in STFT representations. (C) Same plots are shown for OEvents. Here, the highest number of misses are observed for CWT, while in SLT and TFPF the percentage of misses is lower.

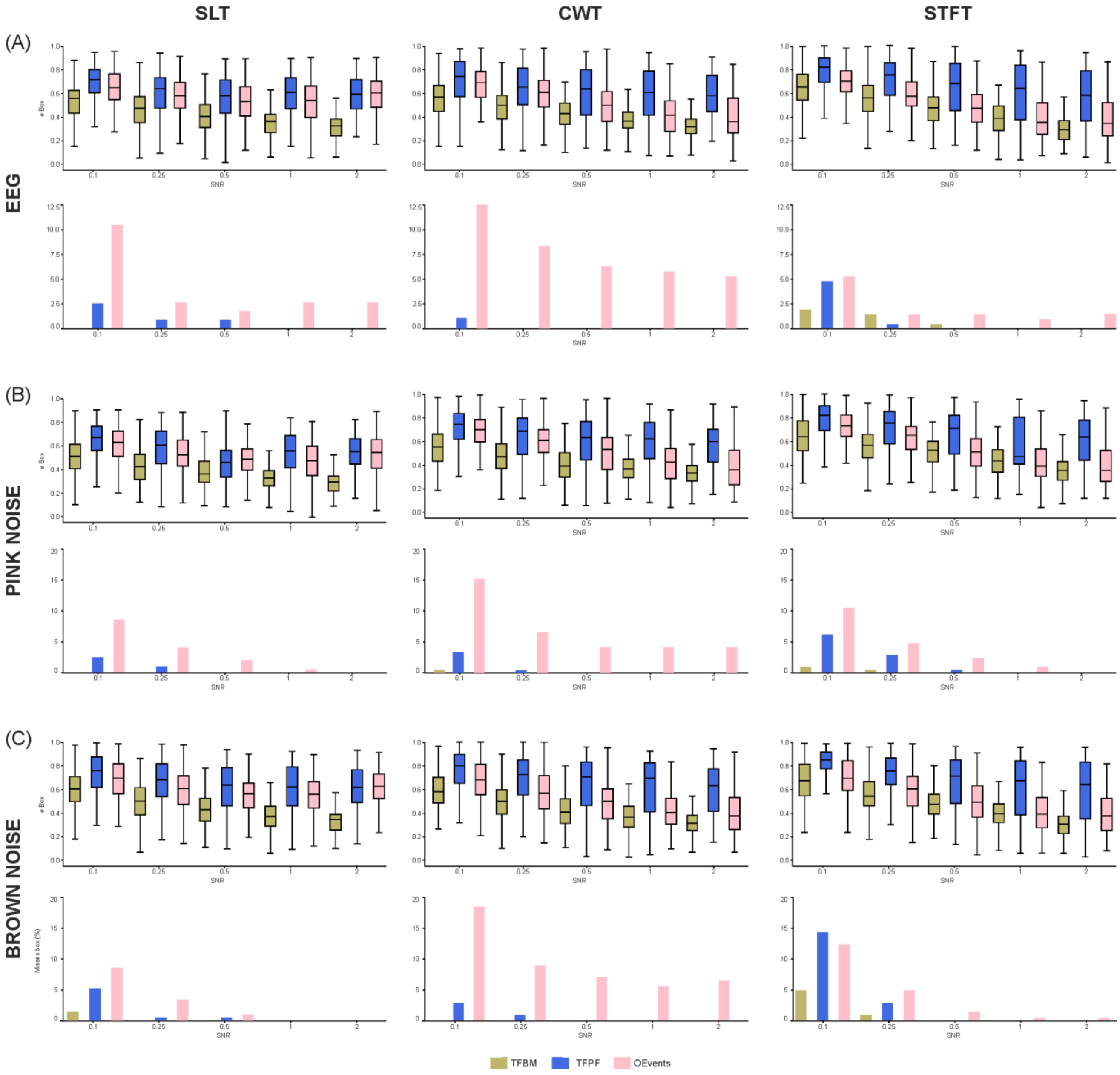

Supplementary Figure 4. The detection performance for atoms buried in different backgrounds (EEG, pink noise, brown noise) for various TFRs (SLT, CWT, STFT). Here, a low power threshold (80%) was used for the same data as Figure 4 of the main text. (A) The box match error (top panes) and the percentage of missed packets (bottom panes) are shown for SLT (left), CWT (center), and STFT (right) for atoms buried in EEG data as background. In (B) and (C) the same evaluation is shown for backgrounds of pink and brown noise respectively.
